# Antiviral candidates for enterovirus 71: targeting viral proteome and stage-specific lifecycle interventions

**DOI:** 10.1186/s12985-025-02966-6

**Published:** 2025-10-31

**Authors:** Jiayi Chen, Tianqi Zhao, Min Ji, Binghui Xia

**Affiliations:** 1 Department of Pharmacy, Traditional Chinese Medicine Hospital of Yangpu District, Shanghai, China; 2 Student 16 Team, Basic Medical College, Naval Medical University, Shanghai, China; 3 Shanghai Yangpu District Mental Health Center, Shanghai, China; 4 Department of Microbiology, Faculty of Naval Medicine, Naval Medical University, Shanghai, China

**Keywords:** Enterovirus 71, Antiviral target, Drugs, Lifecycle, Virus components

## Abstract

Enterovirus 71 (EV71) is a pathogen of concern, especially after its reemergence in most parts of Asia. This virus can lead to severe neurological complications and death, particularly in infants. There are no approved antivirals to prevent or treat EV71 infections, or valid vaccines. EV71 relies on precise virus-host interactions that take place during the viral life cycle, and interference with these offers numerous targets for antiviral strategies that are explored herein. We highlight known antiviral candidates and also alternative avenues to develop novel drugs.

## Introduction

Enteroviruses belong to the Enterovirus genus in the family Picornaviridae [1] and cause several clinical disorders that include neurological diseases. In the genus Enterovirus, enterovirus 71 (EV71) is one of the serotypes in the enterovirus A species (EV-A) [2] that only infects humans. EV71 is further divided into eight genotypes (A to H); B and C have five subtypes each (B1-B5 and C1-C5, respectively), where C1 and C4 are the most prevalent globally [3, 4].

Enterovirus 71 (EV71) was first described in 1974 [5] after analysis of samples collected in California over the previous four years from patients with disease of the Central Nervous System. The authors isolated the strains and presented evidence of their human origin, but the virus was circulating in the Netherlands as early as 1963 [6]. Infection by enterovirus A71 (EV71) can be asymptomatic, or it can result in a mild influenza-like illness. However, EV71 is responsible for major outbreaks of hand, foot, and mouth disease (HFMD) affecting young children, which can result in massive hospitalizations. EV71 can also cause serious neurological problems, from aseptic meningitis to acute flaccid paralysis or encephalitis [7, 8]. Indeed, EV71 is very neurotropic and highly transmissible [7, 9]. Since the initial epidemic in California in 1969, the late twentieth century has seen outbreaks in New York, several European countries and Brazil. Over the last 25 years, there have been HFMD outbreaks also in several Asia-Pacific countries [10] with mortality that typically ranged from < 0.5 to 19% [11, 12]. The unprecedented increase in the number of EV71 infections has been attributed to genotypes B and C. Among the Asia-Pacific countries, China has had the most number of EV71-associated HFMD outbreaks, with 13.7 million HFMD cases between 2008 and 2015, and 3322 deaths [13, 14].

EV71 is transmitted predominantly via the oral-fecal route [15, 16] but also through saliva [17]. Despite this, there are no antivirals available for prevention or treatment. Three vaccines that used inactivated whole viruses have been approved in China but they do not offer broad-spectrum protection against all EV71 strains [18–20], and therefore urgent development of effective countermeasures is required.

**Fig. 1:**
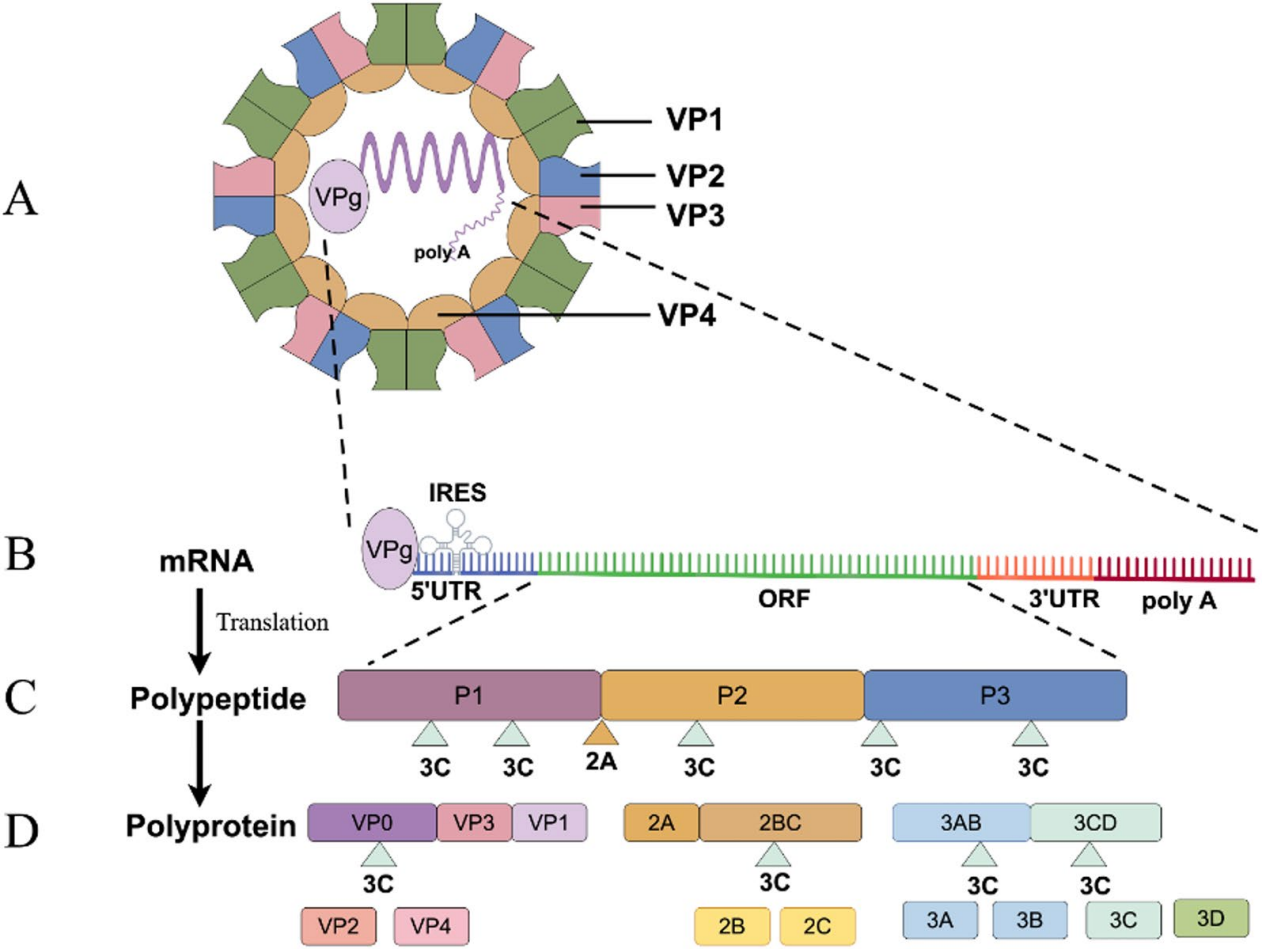
The structure of Enterovirus 71

EV71 has a positive-sense, single-stranded RNA genome encapsidated in a non-enveloped icosahedral virion of about 25 nm in diameter (Fig. 1A). The viral genome (~ 7500 bases long) has a single open reading frame (ORF) flanked by conserved 5’ and 3’ untranslated regions (UTRs) (Fig. 1B). These UTRs have a covalently bound genome-linked viral protein (VPg) (at the 5’ end) and a poly A tail (at the 3’ end) [21]. The 5’ untranslated region (UTRs) of the genome (5’UTR) in EV71 is very conserved, and contains an internal ribosome entry site (IRES) that recruits ribosomes internally, driving the synthesis of the proteome [22]. The central ORF encodes a large polyprotein which can be subdivided into three precursors (P1 to P3) (Fig. 1C). The polyprotein is cleaved by viral proteases into four structural proteins (VP1-VP4, derived from P1), nonstructural proteins 2 A-2 C (derived from P2) and nonstructural proteins 3 A-3D (derived from P3) (Fig. 1D) [23]. See details in Table 1.

The non-enveloped, icosahedral EV71 viral particle has a diameter of ~ 20–30 nm and contains a single positive-sense RNA genome of about 7.4 kb. The EV71 capsid consists of four viral proteins (VP1-VP4), with VP1, VP2, and VP3 exposed on the surface and VP4 embedded inside. The genome features a central open reading frame, conserved non-coding regions at both ends, a covalently bound small protein virus genome-linked protein (VPg) at the 5’ end, and a poly A tail at the 3’ end. The RNA coding region is divided into three subregions: P1, P2, and P3. The P1 region encodes four viral structural proteins (VP1-VP4), while the P2 and P3 regions encode seven nonstructural proteins (2 A-2 C, 3 A-3D).

**Table 1:** The main characteristics and functions of structural and non-structural proteins of EV71

Categories	Protein	Approximate number of amino acids	Characteristics	Functions
Structural Proteins	VP1	297	• The most exposed protein in the capsid • Contains the canyon and pocket factor	• Important for receptor binding, viral entry and virion assembly [24] • Important determinant of EV71 immunogenicity and virulence [24, 25]
	VP2	254	Exposed on the surface of the viral capsid	Contains antibody recognition sites [26]
	VP3	245	Exposed on the surface of the viral capsid	Contains epitopes for neutralising antibodies [27]
	VP4	69	Embedded inside the capsid	Epitope reguired for viral infection and confers stability to the capsid [28]
Nonstructural Proteins	2 A	150	Has a proteinase active site	• Hydrolyzes the peptide bond between P1 and P2 [29] • Inhibits the expression of host cell protein and induces cell apoptosis [30]
	2B	99	Consists two transmembrane helical regions HR1 and HR2	Directly images the mitochondrial membrane potential and induces apoptosis [31]
	2 C	329	Contains ATPase activity	Important for cellular membrane rearrangement and genome replication [32]
	3 A	87	Has a C-terminal transmembrane helix which is responsible for membrane association	Important for viral replication [33]
	3B	22	A primer to initiate RNA synthesis of 3D^pol^	Important for viral replication [34]
	3 C	183	A multifunctional protease	Cuts off the connecting site of P2-P3, degrades DNA repair enzyme, and induces host cell apoptosis [35]
	3D	462	An RNA dependent RNA polymerase (RdRp) with a nuclear localization signal	Responsible for RNA genome replication [36]

## Protein components of EV71

### Structural proteins

The virus capsid comprises 60 identical subunits (protomers) arranged as 12 pentamers, where each protomer has one copy of the four structural viral proteins VP1-VP4 [37]; VP1, VP2 and VP3 are exposed on the surface whereas VP4 is facing the inside of the capsid [38]. Thus, VP1-VP3 are the primary antigenic determinants of the capsid, and since the center of the pentamer is formed by five protruding VP1 proteins, these represent the main antigen and determine the serotype [39]. In enteroviruses, VP1, VP2 and VP3 have β-sandwich “jelly-roll” folds [38, 40] and form a depression around an icosahedral five-fold axis called the “canyon” [41], where receptors that have an immunoglobulin-like fold bind to [42].

### Nonstructural proteins

*P2-derived proteins.* The non-structural proteins originate from precursors P2 (2 A-2 C) and P3 (3 A-3D) (Fig. 1C-D). The EV71 2 A (150 residues) is a cysteine protease (2A^pro^) with a chymotrypsin-like fold with an active site that consists of a catalytic triad formed by C110A, H21 and D39 [30, 43]. 2A^pro^ mediates the initial step of polyprotein processing, cleaving between the C-terminus of VP1 and its own N-terminus (‘2A’ in Fig. 1C) [44, 45], and hijacks host cell gene expression by cleaving eukaryotic initiation factor 4G (eIF4G) [30], whereas 3C^pro^ (see below) performs subsequent polyprotein proteolysis [35].

2B is a membrane protein of 99 amino acids with two transmembrane helical domains that form ion channels critical for virion release [31] and may release calcium into the cytoplasm inducing apoptosis [46].

Lastly, 2 C protein mainly functions as an NTPase, where ATP hydrolysis helps unwind RNA. The crystal structure [32] contains an adenosine triphosphatase (ATPase) domain that belongs to SF3 helicases of the AAA + ATPase superfamily, a cysteine-rich zinc finger and a C-terminal α-helix involved in homo-oligomerization via interaction with a deep pocket formed between the ATPase and the zinc finger domains of a neighboring monomer. Thus, antivirals may be targeting its enzymatic activity and its reliance on self-oligomerization. Additionally, the N-terminal amino acids (~ 50 residues) have potential membrane-binding activity and are involved in membrane remodeling and in the formation of the viral replication complex, where 2 C associates with host reticulon 3 and with the viral double-stranded RNA [47].

*P3-derived proteins.* The 3 A protein (87 amino acids long) also has a C-terminal transmembrane helix which is responsible for membrane association [48], whereas 3B is a ~ 20 residues long peptide known as VPg (virus genome-linked protein) (Fig. 1B) which also binds at the bottom of the palm domain of the 3D^pol^ molecule (see below) [34]. 3 A, together with the host phosphatidylinositol 4-kinase IIIβ (PI4KB), acyl-coenzyme A binding domain containing 3 (ACBD3) and oxysterol-binding protein (OSBP) are involved in the formation of replication organelles [49], which requires the hijacking of host lipid homeostasis. Indeed, 3 A recruits PI4KB via the Golgi-resident ACBD3 protein, creating a phosphatidylinositol 4-phosphate (PI4P)-rich microenvironment, in turn recruiting 3D^pol^ to replication sites where it catalyzes the synthesis of RNA [50]. The structure of this ternary complex is still unresolved, but some structural data of individual components and their interactions are available. For instance, low resolution SAXS-based hybrid structures of PI4KB and ACBD3 and their association have been reported [51]. Furthermore, the structure of the complex formed by ACBD3 GOLD domain and enteroviral 3 A proteins has been determined, e.g., in EV71 3 A (PDB ID: 6HLW), revealing critical interaction motifs [52]. Additionally, PI4KB and ACBD3 have been solved by solution NMR (PDB ID: 2N73), with insights into their interface [53].

The availability of these data suggests that this complex may be targeted for antiviral development. Interestingly, compound N373, which selectively inhibits PI4KB, resulted in resistant mutants localized in protein 3 A [54], suggesting that PI4KB plays a role in the interaction or replication mechanism involving protein 3 A. This is a challenge for identifying drug binding sites in the viral proteome by the method of obtaining revertant mutants; these mutants may appear in viral proteins even though the actual target is a host protein. Therefore, when developing antiviral drugs, it is essential to consider the interplay between viral and host proteins to ensure efficacy and specificity.

The precursor fusion protein 3CD (Fig. 1D) degrades DNA repair enzymes and induces host cell apoptosis [35] and most importantly, leads to formation of 3C^pro^ and 3D^pol^. Similar to other picornaviruses, EV71 3C^pro^ (183 residues) forms a chymoprotease-like fold with two topologically equivalent antiparallel six-stranded β-barrel domains [55]. Located between the two, there is a long shallow groove for substrate binding, but in EV71 this site is very exposed to the solvent. 3C^pro^ cuts itself from the P3 precursor and cuts P2 and P3 to form the final non-structural proteins (see ‘3 C’ in Fig. 1C-D) or the precursor fusion 3CD [56]. 3C^pro^ contains a surface β-ribbon loop, at the base of which Gly123 and His133 form a flexible hinge important in the proteolytic activity [55]. 3C^pro^ also has RNA binding ability and is involved in replication [57].

Lastly, 3D protein (3D^pol^, 462 amino acids) is an RNA-dependent RNA polymerase (RdRp) critical for RNA replication [34]. 3D polymerase is activated by the proteolytic cleavage of the 3CD precursor protein. This activation mechanism is conserved among picornaviruses, even when the 3D protein does not start with a glycine residue [58]. Viral replication complexes are associated with host cell membranes, particularly the endoplasmic reticulum (ER). Recruitment of 3D protein to these membranes involves interactions mediated by the negative charge of the membrane and the 3AB protein [59]. The latter facilitates membrane remodeling and formation of double-membrane vesicles [60]. The 3D protein contains three domains: RNA-binding, catalytic (responsible for RNA synthesis) and inhibitory (which regulates polymerase activity) [61]. Inhibitors targeting the 3D polymerase, discussed later, represent a promising strategy for antiviral therapy, as they can effectively block viral replication [62]. Overall, the 3D protein is central to the replication cycle of EV71, making it a key target for both mechanistic studies and therapeutic interventions [63].

Because of their enzymatic activity, 2 A, 3 C and 3D proteins have been the target for several antivirals. In contrast, membrane-associated 2B, 2 C and 3 A, and intermediates 2BC and 3AB, which are involved in replicating organelle formation have a limited number of drugs reported. This is clearly limited by the lack of structural information for these membrane-associated proteins. Channel activity (2B) and membrane remodeling and oligomerization (2 C and 3 A) should be explored further as targets by using platforms such as cryo-EM or solid state NMR.

## Lifecycle of EV71

**Fig. 2:**
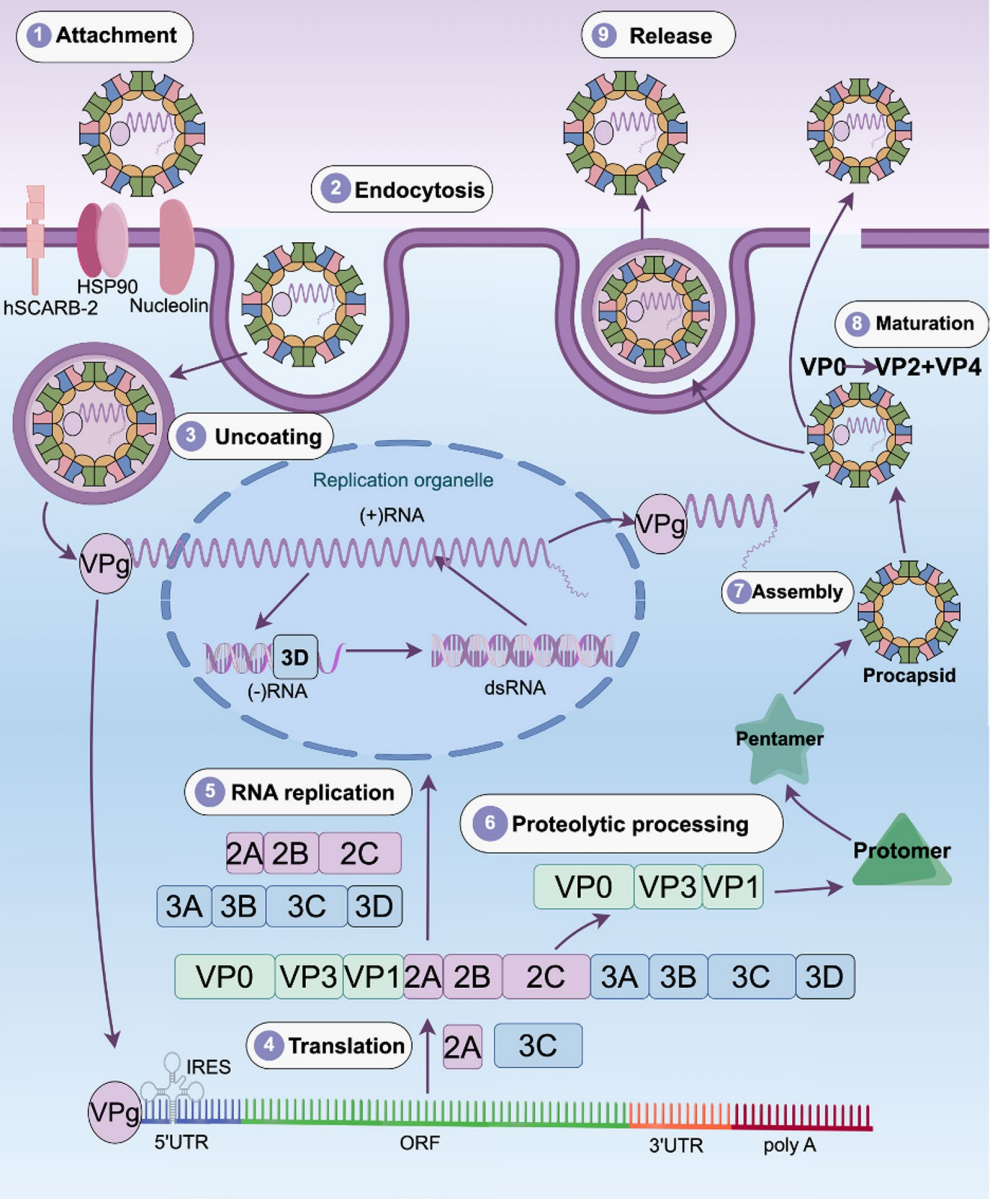
Schematic overview of Enterovirus 71 lifecycle

Virus entry into susceptible host cells starts with attachment to the cell surface and receptor binding (see Fig. 2) to one of the several human receptors identified, e.g., the human scavenger receptor class B member 2 (SCARB2) [64], human P-selectin glycoprotein ligand-1 (PSGL-1) [65], sialic-acid-linked glycan [66], human annexin 2 protein [67] and heparan sulfate glycosaminoglycan [68]. Binding is followed by endocytosis which varies among serotypes and cell types [69]. Binding of receptors to the canyon of the capsid causes expulsion of a hydrophobic “pocket factor” (a lipid molecule) which signals genome release into the cytoplasm [70] through a proteinaceous pore spanning the endosomal membrane [71]. In EV71, this requires both receptor binding and acidification of the endosomal lumen [72, 73]. Upon cytoplasmic entry, RNA is translated into a single polyprotein, and serves as a template for genome replication by 3D^pol^. The newly generated RNA either replicates or forms new virions. After an empty procapsid is formed, RNA is enveloped within the procapsid and triggers cleavage between VP2 and VP4 (Fig. 1D), thus generating mature infectious virus particles [74]. The virion can exit the cells via cell lysis but also through subversion of the autophagic machinery (Fig. 2). Overall, the life cycle of EV71 and enteroviruses in general offer several targets for antivirals [75]. For example, picornavirus genome delivery into the cytoplasm is facilitated by a mechanism previously implicated in the clearance of intracellular bacteria. Indeed, a host lipid-modifying enzyme, adipose-specific phospholipase A2 (PLA2G16), prevents the autophagic degradation of permeated endosomes [76], and therefore PLA2G16 is a possible drug target, as are other host enzymes that regulate autophagy, which are controlled by EV71 and other enteroviruses [77, 78].

The present review focuses on novel antiviral candidates reported in approximately the last five years that target parts of the EV71 life cycle. Inhibitors with specific targets are discussed, while those with unclear targets are tabulated.

EV71 binds to one or more cell surface receptors, triggering viral endocytosis. The virus particles then transport to endosome and release the genome through endosomal membrane holes. Upon cytoplasmic entry, RNA is translated into four structural proteins (VP1-VP4) and seven non-structural proteins (2 A-2 C, 3 A-3D). Simultaneously, RNA serves as a template for genome replication under 3D^pol^ action. The newly generated RNA either replicates or forms new virions. The structural proteins further form an empty capsid. Then RNA is enveloped within the Procapsid and generate mature infectious virus particles. Finally, progeny viruses are released.

## Virus-directed antivirals

**Fig. 3:**
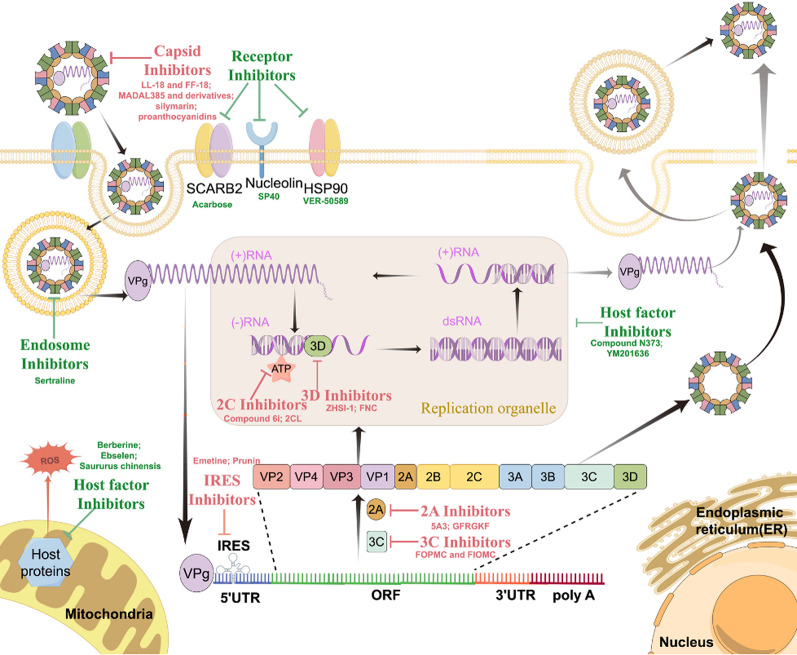
Schematic diagram of antivirals for EV71 with different targets

Antivirals for EV71 can be directed at the virus or at the host (Fig. 3). This section will describe those directed at the virus, which are also shown in Table 2. VP1, 2 A, 3 C and 3D are considered to be the most promising targets since their structures are known.

Antivirals targeting viral components and host cells are presented separately with different colored arrow lines. Virus-directed antivirals are presented in red color, while host-directed antivirals are presented in green color. The most promising antiviral candidates for each category have been selected and presented in the figure.

### Inhibitors targeting VP1 proteins

In picornaviruses, antivirals that target the canyon formed by VP1 can show high affinity [79]. In EV71, this canyon is shallower than that in other enteroviruses and VP1 shows high variability which can result in fast development of resistance. However, highly conserved residues, especially Arg and Pro, have been identified at the intersection between the loops and the strands in the β-barrel in VP1 sequences from all enteroviruses [80, 81]. Targeting these should not only slow down resistance but also lead to pan-enteroviral inhibitors. In general, the capsid has been targeted by peptides, synthetic compounds or natural compounds.

#### Peptides

Although therapeutic peptides have poor in vivo stability and are attacked by enzymes, they have lower off-target toxicity and lower emergence of resistance compared to small drugs (see review in [82]). Historically, lactoferrin—an 80-kDa iron-binding glycoprotein—is one of the earliest EV71 inhibitors: both bovine and human forms bind the capsid protein VP1 and also cellular heparan sulfate receptors, thus inhibiting viral adsorption to cells [83, 84]. This dual binding mechanism was confirmed by ELISA-based binding assays demonstrating lactoferrin-VP1 interaction, which could be competitively blocked by anti-VP1 antibodies [84].

Since proteins and peptides have low membrane permeability, they mostly target the capsid. Other inhibitors are antimicrobial peptides (AMPs), which are part of the innate immune system [85]. For example, cathelicidin (the mature and processed form of cathelicidin in humans is LL-37) is effective against enveloped viruses, but also against non-enveloped ones [86]. In EV71, LL-37 directly inhibited viral binding to cells and inhibited EV71 replication by regulating the antiviral immune response increasing basal interferon-β (IFN-β) expression [87]. Shortening peptides results in general in better bioavailability [88]. Thus, removal of first and last five amino acids of LL-37 produced peptides LL-18 and FF-18 [89]. These 27-residue peptide analogues showed more potency than the parent peptide LL-37 and were found to directly bind EV71 virus particles at the viral canyon region, blocking virus-receptor interactions and inhibiting viral uncoating. This was confirmed by monitoring the changes in the amide region of the peptide by NMR after exposure to the virus and co-immunoprecipitation of the peptides with capsid proteins VP1-VP3. Interestingly, prevented genome release after uncoating and resistance was not observed even after multiple passages, which suggests that mutations that prevent peptide binding would also be deleterious for viral stability [90].

Peptides may also be derived from the VP1 capsid protein of EV71. For example, 95 overlapping 15-mers spanning VP1 were tested, and some inhibited EV71 [91]. One of these peptides, SP81, showed direct virus inactivation of EV71 with an IC_50_ of 8.076 µM, supporting its direct binding to the viral surface [92] although whether VP1 is the actual target remains known.

#### Synthetic compounds

Early efforts yielded compounds like pleconaril, which demonstrated efficacy against other enteroviruses, but activity against EV71 was poor, possibly because insufficient binding to the viral canyon [93, 94]. In contrast, vapendavir forms hydrogen bonds with VP1 residues, e.g., Asp112/Ile113, has anti-EV71 activity and is in clinical development for picornavirus infections [94].

Concurrently, significant progress has been made with novel chemotypes. Rivero-Buceta et al. [95] previously reported a family of tri-and tetrapodal Trp derivatives with prototype AL-385 that inhibited EV71 [96]. AL-385 was found to bind the 5-fold axis of the viral capsid using cryo-EM methods and showed inhibition of EV71 at nM concentrations [97]. By simplifying the scaffold, the same authors identified a second family of EV71 entry inhibitors [98], where two compounds, AL-470 and AL-471, interact with the five fold axis of the capsid (VP1 K244 and Y245). Further, SAR studies developed a new compound based on the same scaffold (a double arylated tetrapodal compound, AL-518) with EC_50_ against EV71 of 40 nM [99]. Further development has resulted in more potent multivalent Trp- and Tyr-containing fullerene hexa-adducts that target both HIV and EV71 [100]. Finally, the insertion of an amphipathic linker in a tetrapodal Trp derivative led to highly potent EV71 inhibitors in clinical isolates [101]. Although these compounds show high affinity and selectivity, and can be optimized with the availability of high resolution structures, it remains to be seen if they can be developed into non-toxic compounds in in vivo studies.

Notably, tanomastat, a repurposed synthetic inhibitor, targets VP1 by binding to its hydrophobic pocket through interactions with key residues and a conserved water molecule, thereby impeding capsid dissociation during viral entry. It exhibits broad-spectrum activity against multiple enterovirus species (A-D) and exerts multi-stage inhibition, including inhibition of viral RNA replication [102].

#### Natural products

Natural products can also target the capsid. A component of Ilex kaushue extracts, 3,4-dicaffeoylquinic acid (3,4-DCQA), showed broad inhibition against different EV71 genotypes by disrupting viral attachment to host cells, specifically targeting E98 and P246 in the 5-fold axis located within VP1, as shown by resistance mutants at these positions. 3,4-DCQA specifically inhibited the attachment of EV71 to the host receptor heparan sulfate (HS), but not to the scavenger receptor class B member 2 (SCARB2) and P-selectin glycoprotein ligand-1 (PSGL1) [103]. This type of interaction may be similar to that observed for the antiparasitic drug suramin (derived from azo dyes) and its sulfated derivative NF449 [104], which also binds near the 5-fold vertex and interferes with attachment to PSGL-1 and heparan sulfate, although in this case VP1 residue 244 was identified as critical for the interaction [105].

Another class of natural compounds that target VP1 are flavonoids such as silymarin or proanthocyanidins (PC). For example, silymarin could inhibit EV71 (IC_50_ of 15 µg/mL) by preventing virus attachment to host cells [106]. The mechanism was proposed to involve binding to the VP1 GH loop [107] which interacts with the uncoating receptor SCARB2 [70]. Similarly, baicalein from *Scutellaria baicalensis* exhibits extracellular virucidal activity against EV71 (IC_50_ = 30.88 µg/mL) by disrupting viral attachment and entry, likely through capsid-binding [106].​​ Proanthocyanidins (PC) may also bind VP1, and intramuscular PC therapy in EV71-infected mice improved survival rates and increased body weight [108]. However, experimental evidence of selectivity, binding sites and affinity of these compounds is lacking, which precludes further development.

### Inhibitors of proteases 2A^pro^ and 3C^pro^

####  2 A protease

To date, 2A^pro^ remains a relatively underexplored target, with no potent 2A^pro^ inhibitors available. Early studies identified the LVLQTM peptide as a classic inhibitor of 2A^pro^, which functions as a pseudosubstrate by binding to the protease’s active site and effectively inhibits EV71 replication [109]. In recent years, research on 2 A has explored its role in immune evasion and inhibitors targeting this role may emerge as a new direction for future antiviral drug development. For example, 2A^pro^ reduces interferon-α receptor 1 (IFNAR1), antagonizing the antiviral activity of IFN-a [110]. It also inhibits the activation of Cyclic GMP-AMP Synthase-Stimulator of interferon genes (cGAS-STING) by targeting tumor necrosis factor receptor-associated factor3 (TRAF3), which helps the virus evade host immune attack [111]. Nevertheless, a region present in viral but not human proteases was identified, and peptides were selected that could bind to that region. Four peptide candidates inhibited 2 A protease activity and the crystal structure of the best peptide bound to 2 A protease was determined [112]. This inhibition may also block the cleavage of antiviral signaling proteins and interferon receptor 1. Additionally, the model peptides can be synthetically modified to resist enzymatic digestion. More recently, etoposide—a semi-synthetic podophyllum derivative—has been identified as a novel 2A^pro^ inhibitor. It targets key residues Y89 and P107 within the protease’s binding pocket, as validated by molecular dynamics simulations and mutagenesis assays. Etoposide exhibits broad-spectrum activity against EV71 genotypes A–C and CVA16, with minimal cytotoxicity even at high concentrations (100 µM). Its established clinical biosafety profile supports potential repurposing as an anti-EV71 agent [113].

####  3 C protease

Rupintrivir (AG-7088) is a peptide [114, 115] that binds to 3 C catalytic pocket via a covalent linkage to Cys147, although clinical trials were unsuccessful [116]. Recent efforts have developed rupintrivir analogues, such as synthetic peptide SG85 [117]. Further modifications of rupintrivir have been attempted using in silico methods [118].

A novel series of peptidomimetic aldehydes was designed to target 3 C protease of EV71 [119]. Not only the compounds showed high antiviral activity but the crystal structure of EV71 3C^pro^ in complex with compound 18p was determined at high resolution (1.2 Å), revealing a covalent linkage to catalytic Cys147 [119].

Two synthetic inhibitors of EV71 3C^pro^, propyl- and isopropyl-substituted 4-iminooxazolidin-2-one moieties (FOPMC and FIOMC) were developed from a previously reported cyanohydrin derivative. However, although these compounds showed inhibitory activity, the binding modes were only shown with in silico docking [120]. Quantitative irreversible tethering (qIT) was used to identify acrylamide fragments that target the active site cysteine of the 3 C protease (3C^pro^) of EV71, where crystal structures of fragments and enzyme were obtained [121].

Additionally, a series of 2-benzoxyl-phenylpyridine derivatives—particularly WY7 —have been identified as potent 3C^pro^ inhibitors. WY7 inhibits EV71 3C^pro^ by inserting into the substrate-binding pocket, forming hydrogen bonds with Thr142 and His161, and disrupting substrate recognition. It also moderately inhibits 3D polymerase, further enhancing antiviral efficacy with an EC_50_ of 11.29 µg/mL in RD cells [122].

Natural products and flavonoids constitute a well-characterized class of 3C^pro^ inhibitors. Quercetin, a widely distributed flavonoid, potently inhibits EV71 3C^pro^ by inserting into its substrate-binding pocket to block substrate recognition, thereby exerting antiviral activity against EV71 [123]. Luteoloside, another flavonoid, inhibits 3C^pro^ in a dose-dependent manner and acts post-attachment to reduce EV71 yields [124]. Fisetin and rutin, two additional flavonoids, directly interact with 3C^pro^ to impair its activity and suppress EV71 plaque formation [125].

Virtual screening of compounds used in traditional Chinese medicine identified salvianolic acid, a polyphenolic compound isolated from *Salvia miltiorrhiza* which inhibited EV71 infection [126]. However, no binding sites were experimentally determined, although in silico docking suggested binding to 3C^pro^.

D-D7 is a peptidomimetic containing 2-pyridone that originally targeted human rhinovirus 3C^pro^ [127]. This drug was repurposed to target the same enzyme in EV71 [127], thereby disrupting polyprotein processing. Future studies should optimize its pharmacokinetics, evaluate combinatorial therapies, and validate efficacy in preclinical models.

### Inhibitors of 2 C protein

2 C is a helicase and an ATPase, and an attractive target for antivirals due to its high conservation [32]. Fluoxetine, a selective serotonin reuptake inhibitor (SSRI) approved by the Food and Drug Administration (FDA), only inhibits species EV B and EV D [128], but the analogue N-(4-fluorobenzyl)-N-(4-methoxyphenyl)furan-2-carboxamide could inhibit all EV strains in the sub-µM range [129]. Escape mutants were found in 2 C near the α2 helix. Using in silico methods the authors proposed a conserved binding pocket in all EVs [129].

In a different study, a quinoline formamide analogue, compound 6i, bound the ATPase domain. This drug could alleviate clinical symptoms in mice when combined with emetine, suggesting binding to 2 C although this was not experimentally validated [130].

Another promising target is the pocket formed between the zinc finger and the ATPase helicase domain, which mediates homo-oligomerization of 2 C [32]. In silico and in vitro testing for 2 C ATPase inhibition identified SJW-2 C-227 as a potent broad-spectrum compound targeting the 2 C:2 C interaction pocket [131]. Peptide 2CL, derived from the 2 C α6 helix, was designed to inhibit 2 C oligomerization by competing with the 2 C α6 helix for binding to a neighboring 2 C deep pocket, demonstrating antiviral properties against EV71 in cells, and improving the survival of mice [132].

The host ubiquitin-proteasome system can be used to degrade viral components. For example, 2 C protein is ubiquitinated by UBE3C, a host E3 ubiquitin ligase, thus restricting EV71 replication. This requires developing UBE3C activators or mimetics with potential broad-spectrum activity against enteroviruses because of 2 C high conservation. However, this strategy risks disrupting host ubiquitination, therefore future efforts should minimize cellular toxicity [133].

### Inhibitors of 3D^pol^

In general, 3D in picornaviruses is highly conserved and is, therefore, an attractive antiviral target [134]. Recent studies have focused on the role of 3D^pol^ beyond RdRp [134], e.g., in interaction with host proteins, where 3D^pol^ can induce cell cycle arrest, regulate host cell translation and induce apoptosis. Inhibitor ZHSI-1 significantly reduced the binding of 3D to lysosomes, inhibiting EV71 replication [135]. Therefore, targeting the lysosome-tethered Ragulator-Rag-3D complex may represent an effective therapeutic strategy. Azvudine/FNC (2’-deoxy-2’-β-fluoro-4’-azidocytidine), a novel cytidine analog originally developed for HIV treatment, binds to 3D^pol^ and incorporates into nascent RNA causing chain termination [136].

Recent advances highlight nucleotide analogs and nucleoside phosphonates as promising candidates targeting the EV71 3D protein. GS-646939, a 4’-cyano-modified C-adenosine triphosphate analog, exhibits exceptional selectivity for picornaviral RdRps, including EV71 [137]. Biochemical studies demonstrate its preferential incorporation over ATP by EV71 3D^pol^, leading to immediate chain termination via steric hindrance during translocation, even under high NTP concentrations. This mechanism contrasts with delayed termination observed in coronaviruses, underscoring its specificity for picornaviruses. In parallel, β-D-xylofuranosyl nucleoside phosphonates, exemplified by adenine derivative 1e, were designed to mimic natural nucleotides [138]. Molecular dynamics simulations suggest binding to the RdRp active site, but activity against enteroviruses is modest (EC_50_ = 16 µM for EV-68), likely due to competitive inhibition and impaired base pairing efficiency [138].

Future development of GS-646939 should focus on optimizing its broad-spectrum efficacy against emerging picornaviruses while minimizing off-target effects, potentially through prodrug engineering or combination therapies. For xylofuranosyl phosphonates, structural modifications to enhance RdRp affinity and nucleobase versatility could improve antiviral potency, particularly against EV71, which remains underexplored in current studies [137, 138]. Both classes face challenges in balancing polymerase selectivity with metabolic stability, necessitating advanced structural modeling and high-throughput screening. Collectively, these agents underscore the therapeutic potential of RdRp-targeted strategies.

### Inhibitors of IRES

The IRES is a relatively new antiviral target, with inhibitors that consist predominantly of natural compounds, some of which repurposed. However, mechanisms of inhibition are poorly characterized, which prevents further development [139]. Historically, several antivirals have demonstrated IRES-targeting activity against EV71, albeit with limited translational progress.​​ Mitoxantrone, initially identified through FRET-based screening, selectively disrupts EV71 IRES-dependent translation by binding to structural RNA motifs [140]. Similarly, the flavonoid apigenin inhibits viral translation initiation by competitively blocking interactions between the IRES and host factors hnRNP A1/A2 [141]. Lycorine indirectly impairs IRES function by arresting viral polyprotein elongation during translation [142].

Recent studies have unveiled novel IRES-targeting strategies. Notably, a binder of 5’-UTR is APOBEC3G, or A3G. This is apolipoprotein B messenger RNA-editing enzyme catalytic polypeptide-like 3G, a cytidine deaminase that inhibits the replication of several viruses. Indeed, A3G inhibited EV71 virus replication, impairing the interaction between the 5’UTR and the host protein poly(C)-binding protein 1 (PCBP1), whereas EV71 overcame A3G suppression through its non-structural protein 2 C, by inducing A3G degradation through the autophagy-lysosome pathway viruses [143]. Compound IMB-Z, a N-phenylbenzamide derivative, was found to increase the levels of intracellular A3G. This effect was unrelated to the enzymatic activity of A3G, but instead, inhibition of EV71 replication was caused by A3G interaction with viral 3D (RdRp) and viral RNA, and packaging into less infective virions. Thus induction of A3G by drugs can be used as a potential therapeutic approach to tackle EV71 infection [144].

Emetine, an antiprotozoal drug, could inhibit EV71 IRES-driven translation in a mouse model [145], whereas small molecule DMA-135 induced a conformational change in the RNA structure and stabilized a ternary complex that inhibited translation [146]. Plant compounds kaempferol or Hsp27 protein inhibitor TDP also can target IRES [147, 148], whereas prunin prevents translation [149, 150].

**Table 2:** Virus-directed antivirals

Target/Mechanism	Classification of antivirals	Name of antivirals	Study stages	Direction of optimization	Study Stages/Experimental model		Reference
					In vitro	In vivo	
VP1	Natural product	Methyl linoleate	The actual target has been identified by molecular docking	Identification of molecular mechanisms	Vero cells	-	[151]
		Ergosterol	The actual target has been identified by molecular docking	Identification of molecular mechanisms	Vero cells	-	[151]
		Mulberroside C	The actual target has been identified by molecular docking	Identification of molecular mechanisms	RD and Vero cells	ICR mice	[152]
		Trans-2-hexenoic acid (THA)	The target has been predicted by molecular docking	Further verification of the antienterovirus spectrum with in vivo evidence and identification of molecular mechanisms	HeLa cells	Kunming mice	[153]
		Rosmarinic acid (RA)	The target has been predicted by molecular docking	Further evaluation of the virucidal activity of RA in combination with other anti-EV71 inhibitors	RD cells	ICR mice	[154]
		Fangchinoline (FAN)	The target has been predicted by viral resistance mutations	Identification of the target and molecular mechanisms	Vero cells	-	[155]
		Silymarin	The actual target has been identified by ELISA competitive binding assay and viral resistance mutations	Further in vivo evaluation	RD cells	-	[107]
		Baicalein	The target has been predicted by molecular docking (virucidal activity)	Improvement of inhibitory activity and acceleration of virucidal effects	RD cells	-	[106]
		Proanthocyanidins (PC)	The actual target has been identified by SPR assay and molecular docking analysis	Further studies in animal models and clinical research	RD and Vero cells	ICR mice	[108]
	Nucleotide sequence	DNA aptamers V7, V11, V21	The actual target has been identified by enzyme linked immunosorbent assay (ELISA)	Combination with nanoparticles, siRNA/miRNA, and chemical drugs to formulate new therapeutic complexes	RD cells	-	[156]
	siRNA with nanoparticles	Se@PEI@siRNA	The actual target has been identified by inhibition test in vitro	Identification of molecular mechanisms	SK-N-SH cells	-	[157]
	Monoclonal antibody (mAb)	cD5	The actual target has been identified by ELISA and Western blot assays	Further studies in animal models and clinical research	RD cells	ICR mice	[158]
	Cathelicidin peptide analogues	LL-18, FF-18	The actual target has been identified by molecular docking and inhibition test in vitro	Further studies in animal models and clinical research	RD and 293 T cells	ICR mice	[90]
	Ilex kaushue extracts	3,4-dicaffeoylquinic acid (3,4-DCQA)	The actual target has been identified by molecular docking and resistant virus selection experiments	Identification of molecular mechanisms and further in vivo evaluation	RD cells	-	[103]
	Tryptophan dendrimers	MADAL385	The actual target has been identified by resistance selection, reverse genetics and cryo-EM	To simplify and reduce the backbone of MADAL385 without affecting the antiviral activity	RD cells	-	[101]
		Tyrosine (Tyr) fullerene 10	The actual target has been identified by molecular docking	Identification of molecular mechanisms	RD cells	-	[99]
	Antiseptic	Povidone-iodine (PVP-I)	The target has been predicted by Western blot analysis	Whether or not PVP-I denatures other viral capsid proteins (VP2 and VP3) or viral genome	Vero, RD, human primary oral fibroblast (HPOF) cells and L929 mouse cells (L-hSCARB2 cells)	-	[159]
	Small molecule	Compound 5 h, 6c	The actual target has been identified by preliminary docking assays	Evaluation of pharmacokinetic parameters and spectrum of activity	RD and MRC-5 cells	-	[160]
		Tanomastat	The actual target has been identified by molecular docking and live virus particle tracking	Further in vivo evaluation and clinical trials	RD and Vero cells	BALB/c mice	[102]
VP3	Chemokine	Platelet factor 4 (PF4) or the CXC chemokine CXCL4	The actual target has been identified by coimmunoprecipitation assay (IP)	Identification of molecular mechanisms	HEK293T and RD cells	Mice	[161]
2 A	Murine mAb	5A3	The actual target has been identified by indirect fluorescent assay, Western blotting and ELISA	Further studies in animal models and clinical research	Vero cells	BALB/c mice	[162]
	Peptide	GFRGKF	The actual target has been identified by crystallography	Further studies in animal models and clinical research	Molecular dynamics simulations	-	[112]
	Natural product	Etoposide	The actual target has been identified by molecular docking, molecular dynamics simulations, and site-directed mutagenesis	Further studies in animal models and clinical research	RD, HEK-293T, and Vero cells	-	[113]
2 C	Pyrazolopyridine-containing small molecules	Compound 7 d, 7 h, 10a, and 19	The actual target has been identified by serial viral passage experiments, coupled with reverse genetics and thermal shift binding assays	The high-resolution X-ray crystal structure of the 2 C protein	RD, A172 and SH-SY5Y cells	-	[163]
	Fluoxetine-derived small molecules	Compound 12b (and analogues 12a, 19b, 19 d)	The actual target has been identified by resistance mutations and thermal shift assays	Structural optimization for enhanced potency/spectrum, mechanistic elucidation via 2 C-inhibitor complex determination, and preclinical safety/efficacy profiling	HeLa, HAPI and BGM cells	-	[129]
	Quinoline formamide analogue	Compound 6i	The actual target has been identified by investigating the dsRNA unwinding in cells	Extensive pre-clinical investigation as a lead compound	RD and Vero cells	Kunming mice	[130]
	Small molecule	SJW-2 C-227	The actual target has been identified by differential scanning fluorimetry (DSF)	To determine a structure of the compound bound to 2 C	RD, HeLa and Vero cells	-	[131]
	Quinoline compound	Compound 6aw	The actual target has been identified by thermal shift assay (TSA)	Further studies in animal models and clinical research	RD cells	-	[164]
	Peptide	2CL	The actual target has been identified by molecular docking	To enhance the antiviral activity, stability, and bioavailability	RD, Vero, Huh7, and 293 T cells	ICR mice	[132]
	E3 ubiquitin ligase	Ubiquitin protein ligase E3C (UBE3C)	The actual target has been identified by Co-IP combined with ubiquitination modification mass spectrometry	The exact combination mode	Vero, human embryonic kidney (HEK293T) cells and human colon cancer (HCT-8) cells	-	[133]
3 C	Macrocyclic peptidomimetic	Compound 4	The actual target has been identified by the crystallographic characterization	Improvements in potency and stability	RD cells	-	[165]
	Acidic polysaccharides	LJ04	The actual target has been identified by inhibition test in vitro	Further investigation of safety and efficacy	MA104 and HM1900 cells	-	[166]
	Michael acceptor	SLQ-4, SLQ-5	The actual target has been identified by molecular docking	Crystallographic study to disclose the precise interaction	RD, 293 T and Vero cells	-	[167]
	Peptidomimetic compound	Rupintrivir (AG-7088)	The actual target has been identified by crystallography	Better water solubility, oral bioavailability and potent activity	RD cells	-	[168]
		Synthetic peptide SG85	The actual target has been identified by X-ray crystallography	Improvement of stability, bioavailability, and activity	RD cells	-	[169]
		Compound 18p	The actual target has been identified by crystallography	Further studies in animal models and clinical research	RD and Vero cells	-	[119]
		FOPMC and FIOMC	The actual target has been identified by molecular docking and molecular dynamics simulations	Further studies in animal models and clinical research	RD cells	-	[120]
	Amino acid fragment	Acrylamide fragments	The actual target has been identified by quantitative irreversible tethering (qIT)	Improvement of specificity and safety	Quantitative irreversible tethering (qIT)	-	[121]
	Small molecule	WY7	The actual target has been identified by inhibition test in vitro and molecular docking	Improvement of inhibitory activity and further in vivo evaluation.	RD cells	-	[122]
	2-pyridone-containing human rhinovirus 3 C protease inhibitor	D-D7	The target has been predicted by crystal structure analysis	Pharmacokinetics and pharmacodynamics of the compounds in animal models	RD cells	-	[127]
	Natural product	Hesperidin	The actual target has been identified by molecular docking	Further in vitro study	Molecular docking	-	[170]
		Salvianolic acid A (SA)	The actual target has been identified by molecular mechanics generalized born surface area (MMGBSA) and steered molecular dynamics (SMD)	To increase the content	RD cells	-	[126]
3D	Pyrimidine analogs	Gemcitabine	The actual target has been identified by RT-PCR and western blot	Further in vivo evaluation	RD cells	BALB/c mice	[171]
		LY2334737	Be used as a gemcitabine prodrug generated by linking gemcitabine covalently to a valproic acid group	Similar dosage as clinical trials	RD cells	BALB/c mice	[171]
		Sofosbuvir	An FDA approved nucleotide analog that already in use for the treatment of hepatitis C infections	To test drug doses to determine the optimum dose suitable for potential clinical trials	RD cells	BALB/c mice	[171]
	Monophosphoramidate adenosine analog prodrug	Remdesivir (GS-5734)	The actual target has not yet been identified	Development of a suitable animal model and combination with other virus entry inhibitors	HeLa cells	-	[172]
	Small molecule	ZHSI-1	The actual target has been identified by biotin tag	The precise mechanism by which ZHSI-1 interacts with 3D polymerase to modulate its function	RD, SK-N-SH, U251 and 293 T cells	C57BL/6J (B6) mice and AG129 mice	[135]
	Cytidine analog	Azvudine/FNC	The actual target has been identified by quantitative real-time reverse transcription-PCR (RT-qPCR), in vitro 3D^pol^ activity, and isothermal titration calorimetry (ITC) experiments	Further studies in animal models and clinical research	RD, 293 T and MDCK cells	ICR mice	[136]
	Nucleotide triphosphate metabolite	GS-646,939	The actual target has been identified by enzyme kinetics, competitive incorporation assays, structural modeling and functional validation	Broad-spectrum antiviral activity against respiratory RNA viruses	-	-	[137]
	Pyrazine-carboxamide ribonucleotide	T-1106 triphosphate	The target has been predicted by molecular dynamics simulations	To optimize nucleoside analogs for broad-spectrum efficacy and reduce resistance and toxicity	RD cells	HSCARB2 mice	[173]
	Nucleoside phosphonate analogues	β-D-Xylofuranosyl adenine nucleoside phosphonate	The actual target has been identified by molecular dynamics (MD) simulations	To overcome steric hindrance during synthesis and refine interactions with RdRp active sites	Cancer cells	-	[138]
3 C and 3D	Natural product	Petroleum ether extract of Tournefortia sibirica L. (PE-TS)	The target has been predicted by compound-target network analysis and molecular docking	To separate effective compounds and uncover theispecific mechanisms	RD cells	BALB/c mice	[174]
IRES	N-phenylbenzamide derivative	IMB-Z	The actual target has been identified by inhibition test in vitro	Further studies in animal models and clinical research	Vero and HeLa cells	-	[175]
	Antiprotozoal drug	Emetine	The actual target has been identified by inhibition test in vitro	To reduce compound toxicity	RD and Vero cells	Kunming mice	[145]
	Small molecule	DMA-135	The actual target has been identified by pull-down experiments in cell culture	To optimize the binding affinity and evaluate the utility as a broad-spectrum inhibitor	Vero and SF268 cells	-	[146]
	Natural product	Kaempferol	The target has been predicted by siRNA knockdown assay	Identification of molecular mechanisms	RD cells	-	[176]
		TDP	The actual target has been identified by comparative proteomic profiling analysis	Further studies in animal models and clinical research	RD cells	-	[147]
		Prunin	The actual target has been identified by viral resistance mutations	More 3D RNA docking studies to affirm the mechanism and the combination of ribavirin and prunin	RD cells	BALB/c mice	[149]
		Licochalcone A (LCA)	The actual target has been identified by luciferase quantitative experiments	Identification of molecular mechanisms	RD and Vero cells	ICR mice	[177]

## Host-directed antivirals

### Inhibitors of entry

The early stages of the virus lifecycle are ideal targets for preventing viral infection because, by this point, the virus has not yet multiplied. Additionally, drugs that target the host can be used as broad spectrum anti-enterovirus inhibitors [178, 179]. Cell surface receptor proteins like hSCARB-2 and HS and host factors associated with replication are promising targets for EV71 treatment. Receptor hSCARB-2 interacts with VP1 and/or VP2 capsid proteins and recognizes all EV71 strains [180]. Acarbose was hypothesized to decrease the intestinal infection of EV71 by binding to this receptor or by inhibiting various glycolic receptors on the cell surface [181].

Cell surface heat shock protein 90 (HSP90) potentially facilitates viral entry, protecting the virus from degradation by the proteasome [180]. Inhibition of HSP90 using geldanamycin (GA) and its analogue 17-allyamino-17-demethoxygeldanamycin (17AAG), or the small molecule VER-50589, effectively countered EV71 both in vitro and in vivo [182].

Nucleolin is a nucleolar phosphoprotein located in fibrillar regions of the nucleolus and on the cell surface [183]. A synthetic peptide from VP1, SP40, could bind to the RNA binding domains (RBDs) of nucleolin, and combination of anti-hSCARB-2 and SP40 led to almost complete viral inhibition (99.5%) [184].

Sertraline, an FDA-approved drug with a long clinical history of use with approval for major depression and obsessive compulsive disorder [185], may be repurposed as a EV71 antiviral. Sertraline is lipophilic and after reaching acidic compartments such as late endosomes and lysosomes, its basic amine groups undergo protonation, thus the drug is trapped in the lysosome and accumulates in acidified compartments, thus sertraline neutralizes the pH level of the endolysosomal route, crucial for entry [186]. The EV71 receptor is localized in the endolysosomal compartments and shuttles to the plasma membrane, therefore sertraline might also target the SCARB2 in the acidic milieu along the endolysosomal pathway. Whether lysosomal-resident proteins, such as ASM and SCARB2, are targets of sertraline merit further investigation. Thus, alkalinization of acidic compartments in host cells is an effective strategy for reducing viral infection and that the lysosome is a viable target organelle for antiviral drug discovery [186]. The lack of inhibitors targeting endocytosis and uncoating steps may be related to the differences in the endocytosis and uncoating steps mediated by different receptors.

### Inhibitors of post-entry stage

Host factors involved in replication complex formation are also potential anti-EV71 targets. For example, phosphatidylinositol-4-kinase IIIβ (PI4KIIIβ) is a key enzyme in the phosphoinositide signalling pathway that is crucial for the replication and survival of various viruses [50]. Inhibitors of this enzyme, N-(4-methyl-5-arylthiazol)−2-amide derivatives, were identified against rhinoviruses with activity also against EV71 [187]. Compound N373 has been reported to inhibit PI4KB, improving the survival and pathology of mice when combined with viral capsid inhibitor G197 [54]. Oxysterol-binding protein (OSBP) binds PI4P inducing the exchange of PI4P in the replicating organelle membrane with cholesterol from the endoplasmic reticulum, which results in increased cholesterol content in the replicating organelle membrane [188, 189]. Also, YM201636 inhibits FYVE finger containing (PIKFYVE) kinase, blocking the endosomal sorting complex required for transport (ESCRT) and disrupting EV71 replication [190]. The small-molecule MPA-CF3, a 10,10’-bis(trifluoromethyl) marinopyrrole A derivative, exerts potent anti-EV71 activity by targeting the host factor coatomer subunit zeta-1 (COPZ1) [191]. Upon binding, this drug disrupts the interaction between COPZ1 and 2 C protein, blocking COPZ1-mediated retrograde transport of 2 C to the ER. This impairs replication organelle formation and viral RNA synthesis. As a host-directed antiviral, MPA-CF3 offers broad-spectrum potential and a high barrier to resistance, but off-target effects and cytotoxicity are a concern [191]. Future studies should focus on optimizing selectivity, validating efficacy in vivo, and exploring combination therapies with direct-acting antivirals.

Other host factors are involved in genome replication. For example, human dihydroorotate dehydrogenase (HsDHODH), a conserved mitochondrial enzyme, supplies pyrimidine nucleotides for RNA synthesis. The small molecule RYL-634 exhibited a strong binding affinity for HsDHODH and shows excellent potency against EV71 (EC_50_ = 4 nM) [192]. Another DHODH inhibitor ML390 inhibited EV71 replication by blocking de novo pyrimidine synthesis, alleviating viral replication and pathogenesis in a mouse infection model [193]. Other natural compounds targeting the post-entry stage have also been identified. Luteolin, a naturally occurring flavonoid, exhibited potent anti-EV71 and coxsackievirus A16 (CA16) activity, as shown by a high-throughput screening assay [194]. Although the target is unknown, time-of-addition studies demonstrated action at the post-attachment stage and inhibition of viral RNA replication. The peptide S78, derived from the host heat shock protein Hsp27, targets the phosphorylation of Hsp27 at Ser78, promoted during infection [195]. This phosphorylation triggers Hsp27 nuclear translocation, inducing the cytosolic redistribution of hnRNP A1. These processes subsequently inhibit viral IRES-mediated translation and replication. Peptide S78 was highly selective and had minimal cytotoxicity, but typical of peptide-based therapeutics, lacked stability and presented problems with intracellular delivery [82]. This approach highlights the potential of modulating host-virus interactions for broad-spectrum antiviral therapies.

Lipid metabolism in the post-entry stage may provide some targets for the design of antiviral drugs [196]. EV71 reportedly exploits host cell lipid β-oxidation to promote its replication, which makes lipid anabolic enzymes potential targets [197]. Drugs like C75 and TOFA inhibit two key enzymes in the fatty acid synthesis pathway, fatty acid synthase (FASN) and acetyl-CoA carboxylase (ACC), respectively, significantly inhibiting EV71 replication, whereas the carnitine palmitoyl transferase 1 (CPT1) inhibitor Etomoxir reduced triglyceride levels (lipid droplets) and inhibited EV71 replication [197]. Recently, FT895 was found to inhibit histone deacetylase 11 (HDAC11), reducing EV71 infection both in vitro and in vivo [198]. HDAC11 has been suggested to support EV71 replication by modulation of lipid droplets and increasing lipid metabolism [198]. In addition, energy generated during lipid metabolism has also been speculated to promote EV71 replication, but the specific mechanism needs to be further studied.

Interference with autotropism and apoptosis, which can be induced by EV71 infection [199], is also an effective way to block virus replication. For example, the PI3K/Akt/mTOR signaling pathway is related to EV71-induced autophagy in mice, where mTOR plays a crucial role facilitating EV71 infection post-entry [77]. Inhibition of mTOR in host cells by a novel Torin2 derivative may be an effective EV71 antiviral strategy [200]. 6-Thioguanine (6-TG), an FDA-approved anticancer agent, inhibits autophagy by targeting host BIRC3, thus suppressing EV71 replication [201]. This drug was highly selective and showed therapeutic potential for EV71-associated diseases.

During EV71-induced apoptosis, ROS generation is observed [202], therefore regulation of the cellular redox environment should protect host cells. Regulation of the Keap1-Nrf2 axis by the natural compound berberine reduced ROS production [203]. ROS generation was also reduced by ebselen, an organoselenium molecule with glutathione oxidase-like activity, thereby decreasing caspase-3 activity and apoptosis [204]. Additionally, a recent study demonstrated that the natural compound magnolol exerts anti-EV71 effects by targeting the Nrf2-SLC7A11-GSH pathway [205]. Magnolol was found to activate Nrf2, leading to increased expression of SLC7A11 and subsequent elevation of intracellular glutathione (GSH) levels. The drug suppressed ROS generation, thereby mitigating EV71-induced oxidative stress and apoptosis [205]. Saucerneol from *Saururus chinensis*, a medicinal herbaceous plant, has anti EV71 activity in Vero cells possibly via regulation of mitochondrial ROS (mROS) generation [206] Table 3.

**Table 3:** Host-directed antivirals

Target/Mechanism	Classification of antivirals	Name of antivirals	Study stages	Direction of optimization	Study Stages/Experimental model		Reference
					In vitro	In vivo	
Host cellular receptors	Peptide	SP81 peptide	The actual target has not yet been identified	Chemical modifications and encapsulation of peptide-based drugs in nanocarriers	RD cells	-	[92]
	Glucosechannel inhibitor	Acarbose	The actual target has been identified by X-ray crystallography	Identification of molecular mechanisms	RD, CCD18-Co, DLD-1, FHC and Neuro-2a cells	ICR mice	[181]
	Geldanamycin analogue	VER-50,589	The actual target has been identified by X-ray crystallography	Structural optimization and reduction of side effects	RD and Vero cells	ICR mice	[182]
	Synthetic peptide	SP40	The binding site has been identified by global docking	Co-crystallization of peptides with the receptors	RD cells	-	[184, 207]
Cell surface glycosaminoglycans (GAGs)	Polynuclear platinum complex (PPC)	TriplatinNC	The actual target has been identified by inhibition test in vitro	Further validation in vivo experiments	RD cells	-	[208]
Inhibit the interaction between viruses and heparan sulfate	Glycosaminoglycan derivative	Fucosylated chondroitin sulfate (FCS)	The actual target has not yet been identified	Identification of molecular mechanisms	Vero cells	-	[209]
Polyamines	Synthetic macrocyclic molecule	Cucurbit[7]uril (CB[7])	The actual target has not yet been identified	Identification of the target and molecular mechanisms	RD cells	-	[210]
Host factors responsible for EV71 RNA replication	Andrographolide Derivatives	ZAF-47	The actual target has not yet been identified	Identification of the target and molecular mechanisms	RD and Vero cells	-	[211]
AKT1, ALB and SRC	Traditional Chinese herbal formula	Changyanning (CYN) Tablets	The actual target has been identified by molecular docking	Further validation in vivo experiments	RD cells	-	[212]
Compete with naturally occurring nucleotides for incorporation into viral RNA	Fluorinated purine analogue	Fludarabine (2 F-ara-A)	The actual target has not yet been identified	Further validation in vivo experiments and reduction of toxicity	Vero, BHK21, U251 MG and HMC3 cells	-	[213]
Lysosomes	Natural product	Enanderinanin J	The actual target has not yet been identified	Identification of the target and molecular mechanisms	Vero cells	-	[214]
Pim1	Synthesized compounds	CX-6258	The actual target has been identified by structure-activity relationship analysis	To determine whether Pim1 directly binds eIF4G or affects eIF4G phosphorylation	RD, HeLa and 293T cells	-	[215]
Alpha-enolase (ENO1)	Glycolysis inhibitor	Dichloroacetic acid (DCA)	The actual target has not yet been identified	Identification of the target and molecular mechanisms	RD cells	-	[216]
Host methyltransferase METTL3	Nucleoside analogues	Compound 2, 11b and 17	The actual target has been identified by molecular docking	Identification of reasons for the marked selectivity of these agents	RD cells	-	[217]
Oxidative stress-mediated ERS/autophagy pathway	Natural product with nanoparticles	Resveratrol-loaded nanoparticles (RES-NPs)	The actual target has not yet been identified	Identification of the target and molecular mechanisms	RD cells	-	[218]
Rho Associated Coiled-Coil Containing Protein Kinase 1(Rock 1)	Kinase inhibitor	GSK269962A	The actual target has been identified by expression of purified proteins in vitro	Identification of molecular mechanisms and evaluation of safety	RD cells	-	[219]
Ameliorate the decrease of caspase-3 and PARP	Natural product	Total astragalosides (ASTs)	The actual target has not yet been identified	Identification of the target and molecular mechanisms	GES-1 cells	-	[220]
Raf-MEK-ERK signaling pathway	Clinically approved B-Raf inhibitor	Vemurafenib	The actual target has been identified by structure-guided chemistry platform	Identification of molecular mechanisms	RD cells	-	[221]
PI3K/AKT/mTOR and NF-κB signaling pathways	Traditional Chinese herbal formula	Pien Tze Huang (PZH)	The actual target has not yet been identified	Identification of the target and molecular mechanisms	RD and Vero cells	ICR mice	[222]
AKT Signaling Pathway	Natural product	Salvianolic Acid B	The actual target has not yet been identified	Identification of the target and molecular mechanisms	HeLa cells	-	[223]
P53 and STAT1 signaling pathway	Polysaccharid	Durvillaea Antarctica Polysaccharide (DAPP)	The actual target has not yet been identified	Identification of the target and molecular mechanisms	Vero cells	-	[224]
Inhibit acid sphingomyelinase (ASM)	Antidepressant	Sertraline	The actual target has not yet been identified	Identification of the target, the molecular mechanisms and antiviral spectrum	RD and HeLa cells	-	[186]
PI4KB inhibitor	Synthesized compound	Compound N373	The actual target has been identified by biochemical assay	Further validation in vivo experiments	RD cells	BALB/c mice	[54]
Disrupt the EV71 replication complex	Inhibitor of PIKFYVE kinase	YM201636	The actual target has been identified by inhibition test in vitro	Identification of molecular mechanisms	RD cells	-	[190]
DHODH inhibitor	Small molecule	RYL-634	The actual target has been identified by chemical rescue experiment	Lead optimization to give more drug-like analogues	RD cells	-	[192]
	Small molecule	ML390	The actual target has been identified by genetic resistance and sequencing efforts	Chemical modifications to reduce plasma protein-binding activity and toxicity	RD, HeLa and Vero cells	ICR mice	[193]
Ser78 phosphorylation site of Hsp27	Peptide	Peptide S78	The actual target has been identified by in vitro and cell-based assays	To Enhance peptide stability, membrane permeability and specificity; reduce off-target effects and toxicity	HEK293 T and RD cells	-	[195]
Inhibit key enzymes in the fatty acid synthesis pathway	Chemical reagent	C75 and TOFA	The actual target has been identified by inhibition test in vitro	Further validation of the specific link in the viral replication cycle that energy is utilized	RD and HUVEC cells	-	[197]
CPT1 inhibitor	Chemical reagent	Etomoxir	The actual target has been identified by inhibition test in vitro	Further validation of the specific link in the viral replication cycle that energy is utilized	RD and HUVEC cells	-	[197]
HDAC11 inhibitor	Analog of N-hydroxy-tetrahydroisoquinoline-7-carboxamide	FT895	The actual target has been identified by bioluminescence resonance energy transfer (BRET) target engagement assay	Identification of molecular mechanisms	HT-29, HeLa and Vero cells	C57BL/6 mice	[198]
PI3K/Akt/mTOR signaling pathway	Torin2 derivative	Compound 11e	The actual target has been identified by molecular modeling study	Better water solubility and potent activity	RD cells	-	[200]
Keap1-Nrf2 axis	Natural product	Berberine (BBR)	The actual target has been identified by inhibition test in vitro	Identification of molecular mechanisms	U251, SK-N-MC and A549 cells	ICR mice	[203]
Reduce ROS generation	Organoselenium molecule	Ebselen	The actual target has been identified by inhibition test in vitro	Identification of the mechanism and the connection between EV71-mediated oxidative damage and ebselen’s antioxidant activity	Vero cells	-	[204]
Nrf2-SLC7A11-GSH pathway	Natural product	Magnolol	The actual target has not yet been identified	The extent to which magnolol modulates levels of innate immune-related genes and proinflammatory factors at the cellular level.	RD and HeLa cells	ICR mice	[205]
Regulate mitochondrial ROS (mROS) generation	Natural product	Saururus chinensis	The actual target has been identified by inhibition test in vitro	Clinical trials and assessment of efficacy and safety	Vero cells	BALB/c mice	[206]
PI3K-AKT	Natural product	Astragaloside IV (AST-IV)	The target has been predicted by network pharmacology	Identification of the targeted binding of AST-IV to PI3K and AKT and further validation in vivo experiments	Normal human gastric epithelial (GES-1) and RD cells	-	[225]
Coatomer subunit zeta-1 (COPZ1)	10,10′-bis(trifluoromethyl) marinopyrrole A derivative	MPA-CF3	The actual target has been identified by quantitative chemo-proteomics	Vivo efficacy evaluation	RD and Vero cells	-	[191]
Inhibit EV71 replication at the early stages of the viral cycle	Scorpion venom antimicrobial peptide derivative	BmKn2-T5	The actual target has not yet been identified	Further understanding of its structural and functional properties, and the design of pharmacologically active peptides with high activity and selectivity	RD and Vero cells	-	[226]
Reduce the expression of baculoviral IAP repeat containing 3 (BIRC3) and attenuate BIRC3-mediated the complete autophagy	A thiopurine drug	6-thioguanine (6-TG)	The actual target has been identified by RNA-sequencing analysis combined with siRNA knockdown, overexpression experiments, and functional validation	To enhance anti-EV71 activity, improve selectivity and reduce cytotoxicity	HT-29 (human colorectal cell lines), HeLa and Vero cells	-	[201]

## Conclusions and perspectives

Targeting host factors has the advantage that emergence of resistance is unlikely and common mechanisms ensure wide applicability to different viruses. However, targeting host factors is inherently dangerous because these targets must remain minimally functional. In contrast, viral targets are in principle more specific to certain strains and have the problem of resistance, especially in RNA viruses. Thus, it is clear that only a combination of treatments can succeed, and only if this treatment is sufficiently short to prevent emergence of resistance. Such combinations, which leverage both viral- and host-targeting agents, can synergistically enhance efficacy while reducing individual drug doses to mitigate toxicity. Effective drugs are available to combat EV71. Possibly the most promising are those that target the capsid, especially when the virus still has not entered the cell. However, since treatment is administered usually after infection, these drugs should be able to penetrate the cell membrane, which is more difficult for peptides. Additionally, peptides are easily hydrolyzed, therefore cyclic peptides, which are resistant to thermal and enzymatic degradation, should be explored. Emerging computational tools can further accelerate the design of such peptides by predicting their stability and binding affinity to viral targets [227].

Higher affinity and lower toxicity can only be optimized when high resolution structures are available by means of X-ray diffraction, solution or solid-state NMR, or cryo EM methods. Even when the target and binding site is known, in the absence of this structural data optimization cannot be performed, and the value of purely in silico methods is questionable.

Beyond the enzymatic activity of some of the proteins in EV71, more attention should be paid to proteins for which we do not have structural data. These proteins have been reported to have channel activity and membrane remodeling activity, which probably depends on oligomerization. These are activities that can be targeted by small molecules, or more likely peptides.

Other alternative compounds such as flavonoids show anti EV71 activity [228] and nanodrug delivery and chemical modifications could be used to enhance bioavailability and potency [170, 229], but detailed structural data is missing.

Translating these candidates to clinical use faces challenges, including the need for age-appropriate formulations, scalable production, and rigorous safety assessment in pediatric populations. In these populations, immature organ function and rapid growth demand tailored dosing strategies.

Notably, given EV71’s propensity to invade the central nervous system, strategies to enhance drug delivery across the blood-brain barrier—such as ligand-functionalized nanocarriers or targeted delivery systems—warrant further exploration to mitigate severe neurological complications. Integrating these advances with a focus on pediatric safety and clinical translatability will be pivotal to advancing EV71 therapeutics.

## Data Availability

No datasets were generated or analysed during the current study.
